# Accurate Prediction of ncRNA-Protein Interactions From the Integration of Sequence and Evolutionary Information

**DOI:** 10.3389/fgene.2018.00458

**Published:** 2018-10-08

**Authors:** Zhao-Hui Zhan, Zhu-Hong You, Li-Ping Li, Yong Zhou, Hai-Cheng Yi

**Affiliations:** ^1^School of Computer Science and Technology, China University of Mining and Technology, Xuzhou, China; ^2^Xinjiang Technical Institute of Physics and Chemistry, Chinese Academy of Sciences, Ürümqi, China

**Keywords:** ncRNA-protein interactions, PSSM, LightGBM, Pseudo-Zernike moments, k-mers

## Abstract

Non-coding RNA (ncRNA) plays a crucial role in numerous biological processes including gene expression and post-transcriptional gene regulation. The biological function of ncRNA is mostly realized by binding with related proteins. Therefore, an accurate understanding of interactions between ncRNA and protein has a significant impact on current biological research. The major challenge at this stage is the waste of a great deal of redundant time and resource consumed on classification in traditional interaction pattern prediction methods. Fortunately, an efficient classifier named LightGBM can solve this difficulty of long time consumption. In this study, we employed LightGBM as the integrated classifier and proposed a novel computational model for predicting ncRNA and protein interactions. More specifically, the pseudo-Zernike Moments and singular value decomposition algorithm are employed to extract the discriminative features from protein and ncRNA sequences. On four widely used datasets RPI369, RPI488, RPI1807, and RPI2241, we evaluated the performance of LGBM and obtained an superior performance with AUC of 0.799, 0.914, 0.989, and 0.762, respectively. The experimental results of 10-fold cross-validation shown that the proposed method performs much better than existing methods in predicting ncRNA-protein interaction patterns, which could be used as a useful tool in proteomics research.

## Introduction

Non-coding RNAs (ncRNAs) are regarded as the “dark matter” in the genome because of their inability in coding proteins. These years, a variety of ncRNA has been discovered by researchers which plays an indispensable role in most processes of vital movements in the field of biology including amino acids transporting, RNA modification and so on (Pan et al., 2016). According to recent research on ncRNA, ncRNA has been proved to be inextricably associated with human diseases and even cancer. For instance, Tian Y et al. have demonstrated that the role of ncRNA in diabetes is emerging significantly since ncRNA is involved in the modulation of 0205 cell mass, insulin synthesis, secretion and signaling (Tian et al., 2018). However, compared to those ncRNAs with known functions in vital processes occurring in living organisms, there is still a large part of ncRNAs whose functions are not yet clear. In order to gain insight into the function of ncRNA, it is essential to determine whether these ncRNAs interact with other proteins which subserve the comprehension of the mechanism behind biological processes involving RNA-Binding proteins(RBPs) (Li and Nagy, 2011). Although reliable models in predicting ncRNA and protein were composed by a large number of experimental analyses such as RBPs (Pan et al., 2017), RPI-Bind (Luo et al., 2017), RNA Compete-S (Cook et al., 2017), there is still a limited number of structural features available in the protein data bank(PDB) about RNA-protein complexes causing these experiments were time-consuming and resource-consuming (Berman et al., 2000) Therefore, researchers focused their attention on predicting interactions between ncRNA and protein only based on sequences which was regarded as a reliable computational approach since the sequences carried enough information required for prediction (Suresh et al., 2015). This sequence-based method can be used to identify potential ncRNA and protein partners in the absence of their structural information during the experiment (Muppirala et al., 2011).

Machine learning provides researchers one of the most cost-effective ways to construct predictive models in an experimental environment where validated training data is available (Muppirala et al., 2011). In Mohammad et al.'s article, they collected motif information and repetitive patterns extracted from validated interactions between RNA and protein with the combination of sequence composition as descriptors to build a RPI prediction model called rpiCOOL by using a random forest classifier (Akbaripour-Elahabad et al., 2016). The random forest classifier is an ensemble of decision trees of which each tree is constructed through training a subset of features that are sampled from the input feature sets randomly (Akbaripour-Elahabad et al., 2016). And in Wang Ying et al.'s article, they proposed a new ncRNA-protein interaction model extended Bayesian classifier which selected valid features by reducing likelihood scores and allowed transparent feature integration during prediction (Wang et al., 2013). After feature extraction, the extracted features were sent to Bayesian classifier for training. Bayesian classifier is one of the most basic statistical classification methods which principle is to calculate the posterior probability of an object by using Bayesian formula, and select the class with the maximum posterior probability as the class to which the object belongs (Cheng et al., 2017). Hai-cheng Yi et al. proposed a computational RPI-SAN model by using the deep-learning stacked auto-encoder network to mine the hidden high-level features from RNA and protein sequences and fed them into a Random forest classifier to predict ncRNA binding proteins (Yi et al., 2018). They further employed Stacked assembling to improve the accuracy of the proposed method (Long et al., 2017; Patel et al., 2017). Including random forests and Bayesian classifiers, these classifiers are traditional classical machine learning classifiers which effectiveness have verified by a large-scale number of experiments (Liu et al., 2016; Wang et al., 2016; Luo and Liu, 2017). However, these traditional classifiers still have much room for improvement in classification performance and time consumption.

In recent years, an improved gradient boosting decision tree classifier named LightGBM has been proposed. LightGBM is a histogram-based decision tree algorithm, which divides continuous feature values into discontinuous feature blocks, and transforms these feature blocks into feature histograms during training (Shi et al., 2018). This LightGBM classifier algorithm had been used to speed up the decision tree building process on GPUs (Graphics Processing Units) and improved its scalability in the article of Huan Zhang et al. (Zhang et al., 2017). In their paper, a large number of experimental data shown that the training speed in constructing decision trees of LightGBM classification algorithm was much faster than general decision tree algorithms with the same classification accuracy (Mitchell and Frank, 2017).

In the field of biology, the discovery of ncRNAs has far exceeded the speed of research on their functions in ncRNA and protein interactions. Therefore, it is urgent to study an efficient prediction tool in the field of ncRNA-protein interactions which is less-time consuming and resource saving. Hence, we applied this efficient LightGBM classifier to large-scale ncRNA and protein interaction prediction and proposed a new machine learning model using sequence-based information named LGBM in this context. More specifically, each sequence of ncRNA is converted into a k-mers sparse matrix and the feature vectors of ncRNA are extracted from the resulting k-mers sparse matrices using the singular value decomposition (SVD). For proteins, based on the evolutionary point mutation model of protein sequences, we converted each protein sequence into a position-specific scoring matrix (PSSM) where the position information and frequency information were contained. Afterwards, each protein sequence was characterized by the feature vector obtained from a transform processing by using the pseudo-Zernike moment (PZM) algorithm. After extracting features of ncRNA and protein, we fed these reprehensive features into LightGBM classifier for classifying learning and predicting interactions between ncRNA and protein. In order to evaluate the predictive performance of the machine learning model, we used a 10-fold cross-validation to reduce overfitting. During the experiment, we employed four benchmark datasets to evaluate the performance of our model which was RPI369, RPI488, RPI1807, and RPI2241, respectively, and compared the prediction results of our model with other advanced models at the present stage. Experimental results indicated that our model LGBM performed well on four datasets above.

## Methods

### Protein feature extraction

In this section, we selected the PZM feature extraction algorithm to extract sequence-based protein feature vectors using PSSM (Maali et al., 2016; Kheirkhah et al., 2017). The PSSM algorithm first integrates the biological evolution information to predict distantly related proteins, and has achieved good performances in protein binding sites and disordered region prediction (Yi et al., 2018). Let *P* be a PSSM matrix as the representative of an arbitrary protein. A matrix *P* consists of *r* rows and 20 columns with the explanation that *r* means the length of the primary sequence of an arbitrary protein while 20 means the quantity of amino acids (Sharma et al., 2013). Based on this, a PSSM matrix is represented as follows:

(1) $$\begin{array}{l} P=\left[\begin{array}{l} p_{1,1}\cdots p_{1,20}\\ \vdots \ddots \vdots \\ p_{r,1}\cdots p_{r,20} \end{array}\right] \end{array}$$

Where *p*_*i, j*_ in *i*_*th*_ row *j*_*th*_ column denotes the relative probability of *j*_*th*_ amino acid at the *i*_*th*_ position of the same protein sequence with which PSSM matrix comes from (Hayat and Khan, 2011). In experiments, the position-specific iterated BLAST (PSI-BLAST) tool was used to transform original protein sequences into PSSM matrices with the parameter *err-value* set to be 0.001.

Then we extracted PZM feature vectors from the resulting PSSM matrices above. The PZM is a statistical feature extraction algorithm that is computationally efficient for using global information to extract features (Haddadnia, 2001). Pseudo-Zernike polynomials are orthogonal sets of complex-valued polynomials defined as follows (Haddadnia et al., 2003):

(2) $$\begin{array}{l} V_{\alpha \beta }\left(x,y\right)=R_{\alpha \beta }\left(\rho \right)\text{exp}\left(j\beta {\text{ tan}}^{-1}\left(\frac{y}{x}\right)\right) \end{array}$$

Where *x*^2^ + *y*^2^ ≤ 1, α ≥ 0, |β| ≤ α and $\rho =\sqrt{x^{2}+y^{2}}$ is the length of the vector from the origin to the pixel (x, y). And the radial polynomials *R*_αβ_ are defined as:

(3) $$\begin{array}{l} R_{\alpha \beta }\left(x,y\right)=\sum_{t=0}^{\alpha -|\beta |}Z_{\alpha ,|\beta |,t}{\left(x^{2}+y^{2}\right)}^{\frac{\alpha -t}{2}} \end{array}$$

Where

(4) $$\begin{array}{l} Z_{\alpha ,|\beta |,t}={\left(-1\right)}^{t}\frac{2\alpha +1-t}{t!\left(\alpha -|\beta |-t\right)!\left(\alpha -|\beta |-t+1\right)!} \end{array}$$

And *R*_α,−β_(ρ) = *R*_α,β_(ρ) Therefore, the Zernike moments of order α with repetition β for a continuous image function *f*(*x, y*) that vanishes outside the unit circle are as follows (Kim and Lee, 2003):

(5) $$\begin{array}{l} M_{\alpha \beta }=\frac{\alpha +1}{\pi }\iint_{x^{2}{+y}^{2}\leq 1}f\left(x,y\right)V_{\alpha \beta }^{*}\left(\rho ,\theta \right)dxdy \end{array}$$

Pseudo-Zernike polynomials are orthogonal and satisfy the following equation:

(6) $$\begin{array}{l} \iint_{x^{2}{+y}^{2}\leq 1}\left[V_{\alpha \beta }^{{}^{*}}\left(x,y\right)\right]\times V_{mn}\left(x,y\right)dxdy=\frac{\pi }{\alpha +1}\delta_{\alpha m}\delta_{\beta n} \end{array}$$

With

(7) $$\begin{array}{l} \delta_{ab}=f\left(x\right)=\{\begin{array}{l} 1,\;a=b\\ 0,otherwise \end{array} \end{array}$$

Hence, based on the derivation of the above formulas, the feature vectors of protein sequences can be represented as follows (Wang Y. et al., 2017):

(8) $$\begin{array}{l} \vec{F}={\left[\left|M_{11}|,|M_{22}|,\cdots \;,|M_{\alpha \beta }\right|\right]}^{T} \end{array}$$

### ncRNA feature extraction

As for ncRNA, we used the SVD algorithm to extract feature vectors from the k-mers sparse matrix represented ncRNA sequences. In the k-mers sparse matrix construction algorithm, we traversed each complete ncRNA sequence (A, C, G, U) stepping one nucleotide at a time, which is considered characteristic of each nucleotide (Yi et al., 2018). After that, the frequency of the combined triplet feature based on 4 nucleotide letters was extracted for each RNA sequence and obtained 4^*k*^ dimensional features (You et al., 2016). Each characteristic value is the normalized frequency of 4-mers nucleotides in the ncRNA sequences, which is AAAA, AAAC … TTTT (Pan et al., 2016). Therefore, we obtained matrices including frequency information, location information and more hidden information represented the ncRNA sequences (Yi et al., 2018).

Furthermore, we used SVD algorithm to decompose k-mers sparse matrix. The *Q* represent the original k-mers sparse matrix from above and there is singular value decomposition as follows:

(9) $$\begin{array}{l} Q=U\Sigma V \end{array}$$

Where the elements of diagonal in Σ represent the singular value of *Q*. It obtained the most information from original matrix *Q*. Consequently, We reconstruct a 1 × 4^*k*^ dimensional vector from *Q* shows as follows:

(10) $$\begin{array}{l} \vec{F}=U \end{array}$$

### LightGBM algorithm

After obtaining potential features of ncRNA and protein calculated from above feature representation approaches, we fed these high-level features into LightGBM classifier to train the prediction scheme for predicting RPIs.

The traditional gradient boosting decision tree (GBDT) algorithm is a widely used machine learning algorithm which ensemble decision trees in an integrated learning model (Ke et al., 2017). This GBDT algorithm learns the decision trees by fitting the negative gradients (Friedman, 2001). In the process of learning decision trees, the most time-consuming and labor-consuming step is to find the best split points (Appel et al., 2013). The traditional GBDT algorithm uses the histogram-based algorithm to store continuous eigenvalues into discrete regions which are used to construct feature histograms during training instead of selecting the best split points (Li et al., 2007). However, with the increase of data volume, the workload of scanning all the data instances to estimate the information gain of all possible split points is increasing which costs time-consuming a lot (Chen and Guestrin, 2016). In order to address the limitation of this problem, an improved algorithm based on GBDT named LightGBM was proposed which improving the accuracy of classification in proposing two new novel techniques called Gradient Based One-side Sampling (GOSS) and Exclusive Feature Bundling (EFB) (Ke et al., 2017).

Through the GOSS algorithm, the problem that no native sample weights in GBDT avoiding hurting the accuracy of the learned model was solved by discarding those data instances with small gradients. Firstly, training instances were sort by their gradients from high to low in order. Second, select top *p* × 100% instances with high gradients and sample *q* percent data instances in the remaining subsets randomly. Let *A* ∪ *B* represents their collection. Hence, the estimated variance gain $\tilde{V_{s}}\left(b\right)$ of splitting feature *s* at point *b* over the subset *A*∪*B* can be define as follows (Ke et al., 2017):

(11) $$\begin{array}{l} \tilde{V_{s}}\left(b\right)=\frac{1}{n}(\frac{{\left(\sum_{x_{i}\in A_{l}}g_{i}+\frac{1-p}{q}\sum_{x_{i}\in B_{l}}g_{i}\right)}^{2}}{n_{l}^{s}\left(b\right)}\\ \;+\frac{{\left(\sum_{x_{i}\in A_{r}}g_{i}+\frac{1-p}{q}\sum_{x_{i}\in B_{r}}g_{i}\right)}^{2}}{n_{r}^{s}\left(b\right)}) \end{array}$$

Where *A*_*l*_ = {*x*_*i*_ ∈ *A*; *x*_*ij*_ ≤ *b*}, *A*_*r*_ = {*x*_*i*_ ∈ *A*; *x*_*ij*_ > *b*}, *B*_*l*_ = {*x*_*i*_ ∈ *B*; *x*_*ij*_ ≤ *b*} and *B*_*r*_ = {*x*_*i*_ ∈ *B*; *x*_*ij*_ > *b*}.

On the second step, the EFB algorithm was used to effectively reduce the number of features by bundling exclusive features into a single feature avoiding hurting the accuracy. By adopting the EFB algorithm, building the same feature histograms from the resulting feature bundles above were available as those from individual features (Meng et al., 2016). Therefore, the complexity of histogram building was reduced from *O*(*#data* × *#feature*) to *O*(*#data* × *#bundle*) since *#bundle*≪*#feature*. First, we used NP-hard to partition features into a smallest number of exclusive bundles just as the graph coloring problem (Zuev, 2015). Second, offsets were added to the original values of feature vectors to merging the features in the same bundle and ensured that the values of the original values can be identified from the resulting feature bundles.

### Evaluation criteria

In this study, we used a 10 - fold cross-validation method to avoid overfitting and guarantee the accuracy of our algorithm of our model which divided the datasets into 10 equal parts randomly. During each training test, one part was taken as the testing dataset, while the remaining nine parts were the training datasets^1^. Therefore, a total of 10 experiments were conducted. To evaluate the performance of our model LGBM, we followed several widely used evaluation criteria including accuracy, sensitivity, specificity, precision, and Matthews Correlation Coefficient(MCC) as follows (Liu and Chen, 2012):

(12) $$\begin{array}{l} Accuracy=\frac{TP+TN}{TP+TN+FP+FN} \end{array}$$

(13) $$\begin{array}{l} Sensitivity=\frac{TP}{TP+FN} \end{array}$$

(14) $$\begin{array}{l} Specificity=\frac{TN}{TN+FP} \end{array}$$

(15) $$\begin{array}{l} Precision=\frac{TP}{TP+FP} \end{array}$$

(16) $$\begin{array}{l} MCC=\frac{TP\times TN-FP\times FN}{\sqrt{\left(TP+FP\right)\left(TP+FN\right)\left(TN+FP\right)\left(TN+FN\right)}} \end{array}$$

where *TP, FP, TN*, and *FN* are respectively interpreted as the number of true positive, false positive, true negative and false negative. The Receiver Operating Characteristic(ROC) curve can be represented as the threshold between *SP* and *SN*, which *x*-ray depicts false positive rate (FPR) while *y*-ray depicts true positive rate (TPR) (Huang et al., 2015). Meanwhile, the AUC is regarded as the area of the graphical under the ROC curve.

### Datasets

To verify the robust and effectiveness of our model LGBM, we selected four ncRNA and protein interactions datasets including RPI369, RPI488, RPI1807, and RPI2241. Among them, the dataset RPI369 and RPI2241 were selected from the databases PRIDB which is a database of ncRNA-protein interfaces calculated from their complexes in the protein data bank (Berman et al., 2000; Wang et al., 2013). RPI2241 is a positive sample set consisting of 2,241 pairs of experimentally verified ncRNA-protein pairs including 2,043 protein chains and 842 ncRNA chains. RPI369 is a subpart of RPI2241 with 369 pairs including 338 protein chains and 332 ncRNA chains which excludes all ncRNA-protein interaction pairs that interact with ribosomal proteins or ribosomal ncRNA in various organisms (Muppirala et al., 2011). For dataset RPI369 and RPI2241, an approximately negative sample dataset was constructed with twice number of pairs by pairing ncRNA and protein sequences after removing the pairs in the positive sample dataset randomly (Wang et al., 2013). RPI488 is a non-redundant lncRPI dataset based on structural complexes which consists of 488 lncRNA-protein pairs, including 245 non-interacting pairs and 243 interacting pairs from shen et.al. (Pan et al., 2016). And RPI488 is smaller than other datasets since there are fewer lncRNA-protein complexes in PDB where ncRNA-protein complexes are destroyed from downstream (Ying et al., 2010). The dataset RPI1807 consists of 1807 positive ncRNA-protein pairs including 1078 ncRNA chains and 1807 protein chains and 1436 negative pairs with 493 ncRNA chains and 1436 Protein chains. It is established by parsing a nucleic acid database (NAD) that provides RNA protein complex data and protein RNA interface data (Yi et al., 2018). The specific composition of these four datasets are described in Table 1.

**Table 1:** The specific composition of four required datasets.

**Datasets**	**Positive pairs**	**Negative pairs**	**The number of proteins**	**The number of ncRNAs**
RPI369	369	369	338	332
RPI488	243	245	247	25
RPI1807	1,807	1,436	1,807	1,078
RPI2241	2,241	943	2,043	842

### Experimental results

**Figure 1:**
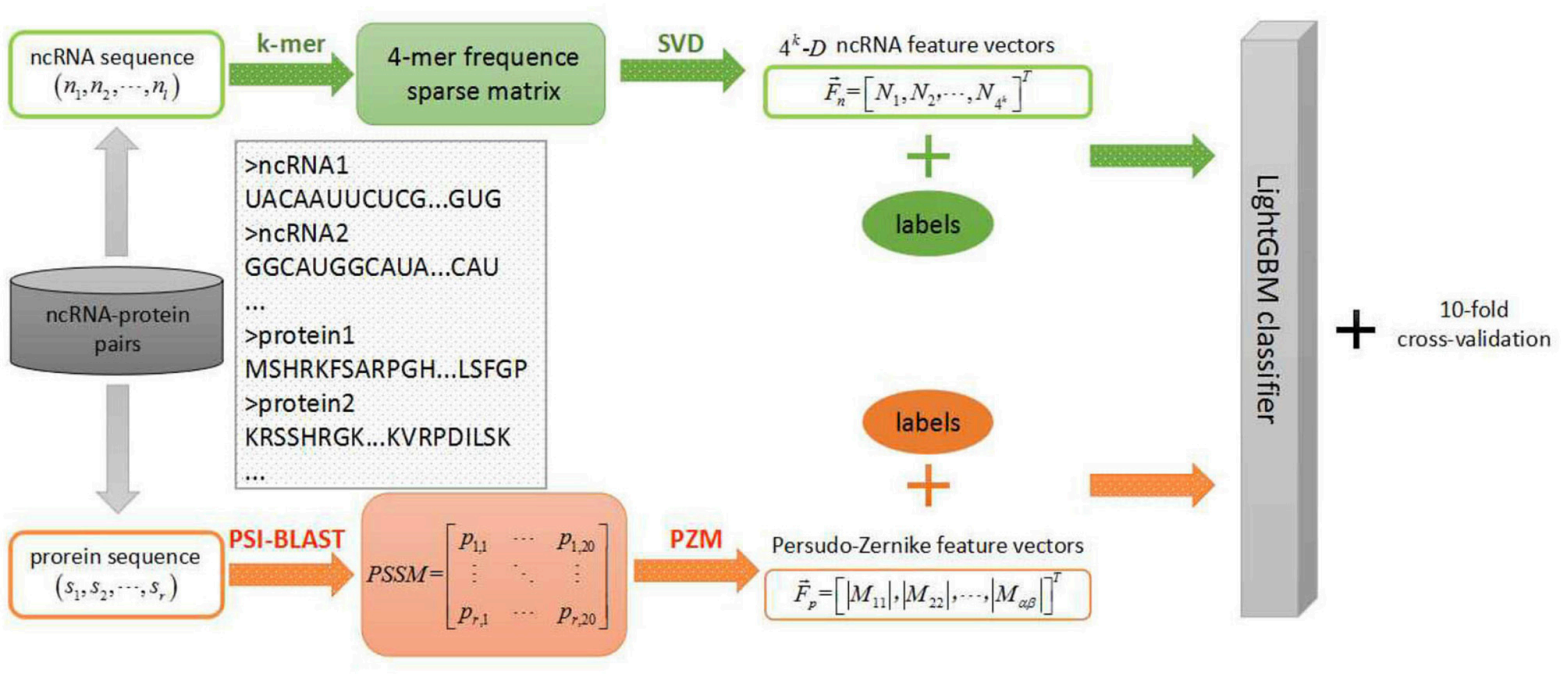
Step-wise work flow for the purposed LGBM machine learning model.

In this study, we proposed a machine learning classification model based on improved gradient boosting decision tree to predict interactions between ncRNA and protein named LGBM which used PSSM and PZM algorithms to extract protein feature vectors and combined k-mers matrices and SVD algorithms to extract RNA feature vectors. The specific steps of the machine learning model are shown in the Figure 1. To verify the performance of the proposed model LGBM, we evaluated the prediction ability of LGBM on datasets RPI369 and RPI488 and had a comparison with the prediction performance of other classifiers under the same feature extraction condition firstly. In addition, we also evaluated the predictive performance of datasets RPI1807 and RPI2241 and compared the prediction results of these two datasets with those of other proposed models in earlier papers.

### Prediction ability of LGBM

In this section, we validated our machine learning model LGBM with 10-fold cross-validation on datasets RPI369 and RPI488 to predicting ncRNA-protein interactions. The 10-fold cross-validation contributed LGBM to avoid over-fitting and had a better performance. As a result, the summary of experimental prediction results under 10-fold cross-validation are shown in Tables 2, 3.

**Table 2:** Ten-fold cross-validation results on dataset RPI369.

**Testing set**	**Accuracy (%)**	**Precision (%)**	**Sensitivity (%)**	**Specificity (%)**	**MCC (%)**
1	75.00	73.17	81.08	68.57	50.12
2	70.83	73.53	67.57	74.29	41.90
3	76.39	76.32	78.28	74.29	52.73
4	76.39	75.68	77.78	75.00	52.80
5	76.39	71.11	88.89	63.89	54.51
6	69.44	65.91	80.56	58.33	39.89
7	73.61	72.97	75.00	72.22	47.24
8	75.00	72.50	80.56	69.44	50.31
9	74.65	71.43	83.33	65.71	49.89
10	70.42	69.23	75.00	65.71	40.91
Average	73.81	72.18	68.75	78.81	48.03

**Table 3:** Ten-fold cross-validation results on dataset RPI488.

**Testing set**	**Accuracy (%)**	**Precision (%)**	**Sensitivity (%)**	**Specificity (%)**	**MCC (%)**
1	91.84	100.0	83.33	100.0	84.76
2	87.76	94.00	77.27	96.30	75.91
3	87.76	88.89	88.89	86.36	75.25
4	95.92	100.0	92.00	100.0	92.15
5	75.51	75.00	60.00	86.21	48.43
6	91.84	91.30	91.30	92.31	83.61
7	93.75	100.0	86.36	100.0	87.99
8	87.50	96.30	83.87	94.12	75.19
9	91.60	95.24	86.96	96.00	83.54
10	91.67	91.67	91.67	91.67	83.83
Average	89.52	93.28	94.30	84.17	79.02

As shown in Tables 2, 3, when LGBM machine learning model was used to predict interactions between ncRNA and protein on dataset RPI369, the mean performance of accuracy, precision, sensitivity, specificity and MCC were 73.81, 72.18, 68.75, 78.81, and 48.03%, respectively. While for dataset RPI488, the mean performance of accuracy, precision, sensitivity, specificity and MCC highly achieved 89.52, 93.28, 94.30, 84.17, and 79.02%, respectively. At the meantime, in 10-fold cross-validation, the accuracy of one validation was even as high as 95.92% while there were other five validations achieved the accuracy of 90%.

The prediction accuracy of LGBM on datasets RPI369 and RPI488 illustrated the feasibility of predicting ncRNA and protein interactions only based on their sequence information. In fact, the protein and ncRNA feature extraction methods can extract more in-depth information hidden in sequences including location, frequency and interaction information into PSSM matrices and k-mers matrices (You et al., 2016). In addition, selecting PZM algorithm to extract feature vectors makes better use of the properties of PZM (Khotanzad and Hong, 1990).

### Comparison between different classifiers

**Figure 2:**
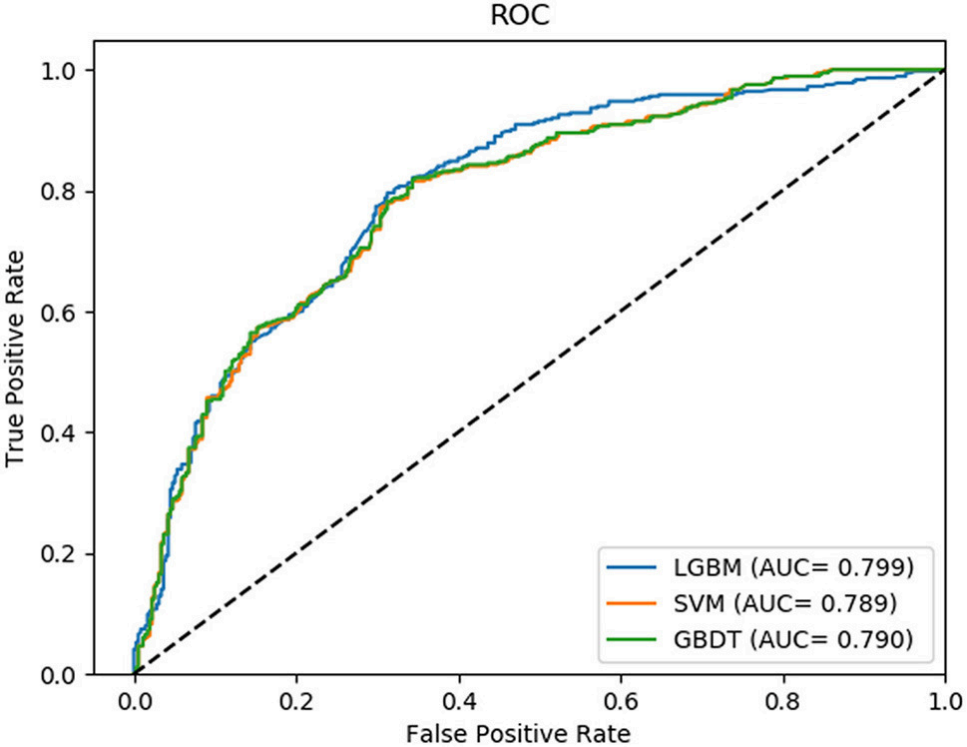
The ROC curve of dataset RPI369 on three classifiers.

**Figure 3:**
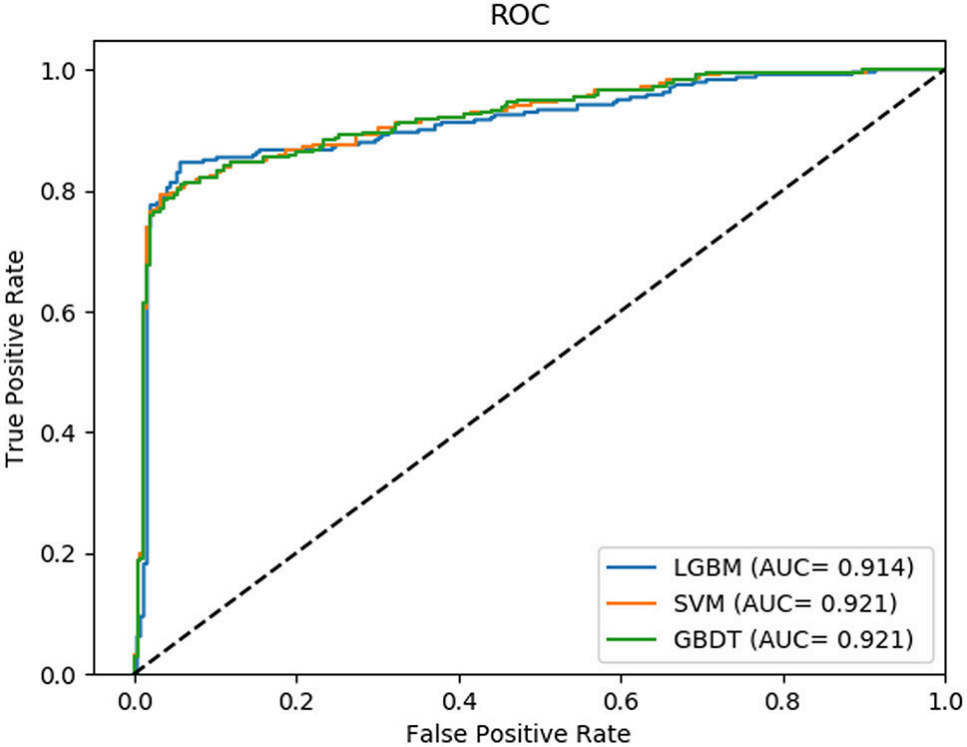
The ROC curve of dataset RPI488 on three classifiers.

In this comparison module, we compared the prediction performance of LightGBM classifier, SVM classifier and traditional gradient boosting decision tree classifier in datasets RPI369 and RPI488 sharing the same feature extraction condition. As a result, the summary of experimental prediction results under 10-fold cross-validation is shown in Table 4 and the corresponding trade-off between false positive rate and true positive rate shown in the receiver operating characteristic (ROC) curve in Figures 2, 3.

**Table 4:** Performance evaluation on different classifiers.

**Dataset**	**Classifier**	**Accuracy (%)**	**Precision (%)**	**Sensitivity (%)**	**Specificity (%)**	**MCC (%)**
RPI369	LGBM	**73.81**	**72.18**	68.75	**78.81**	**48.03**
	SVM	71.60	71.70	**70.74**	72.51	43.62
	GBDT	71.74	71.79	**70.74**	72.79	43.90
RPI488	LGBM	**89.52**	**93.28**	**94.30**	**84.17**	**79.02**
	SVM	86.22	88.62	89.86	82.27	72.44
	GBDT	86.01	88.54	89.86	81.81	72.04

*The bold value indicates this measure performance is the best among the compared methods*.

As can be seen from Table 4, the LightGBM classifier achieved an accuracy of 73.81% in predicting interactions between ncRNA and protein in dataset RPI369, which was higher than 71.60% of SVM classifier and 71.74% of traditional GBDT classifier. And as for precision, sensitivity and MCC except specificity, the LightGBM classifier also had a better performance with exact percent of 72.18, 78.81, and 48.03% respectively, while 71.70, 72.51, and 43.62% under SVM classifier and 71.79, 72.79, and 43.90% under traditional GBDT classifier. For dataset RPI488, whether accuracy, precision and sensitivity or specificity and MCC, LightGBM classifier performed better than the other two classifiers with the exact results of 89.52, 93.28, 84.17, 94.30, and 79.02%, respectively. That is to say, under the evaluation of each evaluation criterion, our LightGBM classifier had a better classification performance than SVM and traditional GBDT classifiers which proved the feasibility and effectiveness of choosing LightGBM classifier to process sequence information in our model LGBM.

The comparison results shown the feasibility and effectiveness of selecting LightGBM as classifier in our model (Zhu et al., 2017). In fact, LightGBM, as an improved gradient boosting decision tree, processing the advantages of reducing the number of features and gaining enough information gain through smaller datasets by EFB and GOSS, is superior to other classifiers in terms of computational speed and memory consumption (Wang et al., 2017).

### Comparison with other existing methods

**Figure 4:**
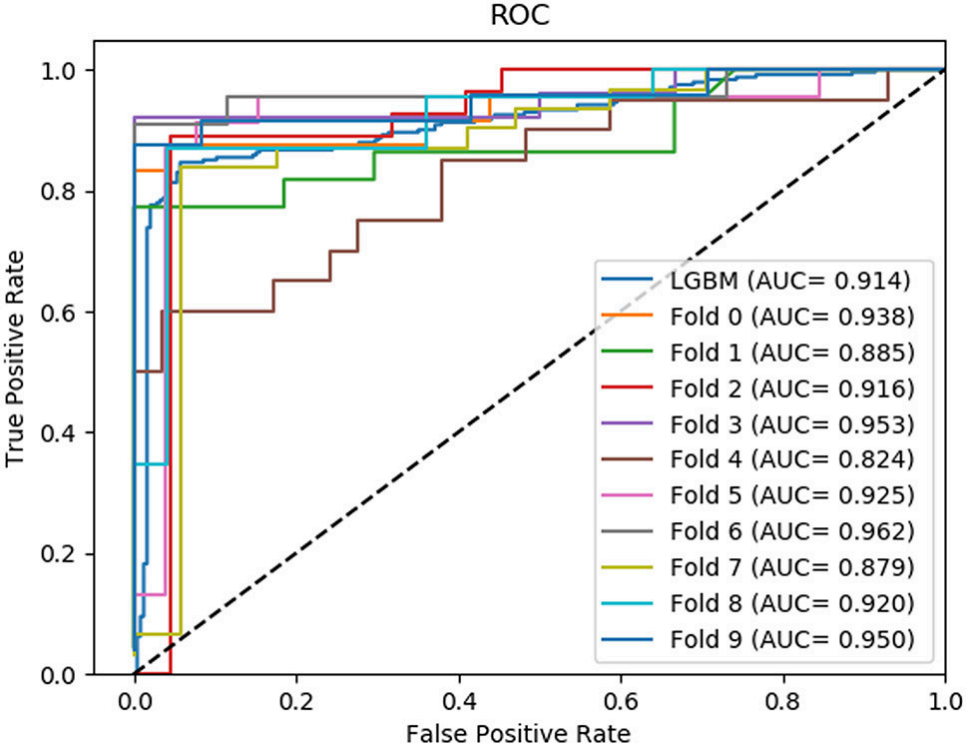
The ROC curve of dataset RPI488 on 10-fold cross- validation.

**Figure 5:**
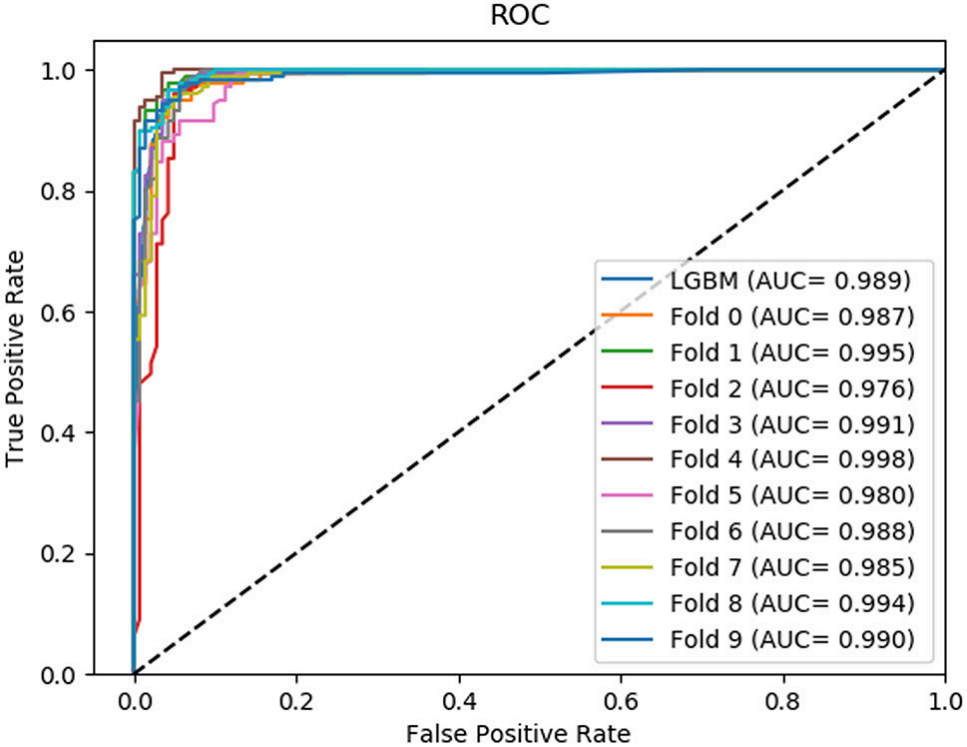
The ROC curve of dataset RPI1807 on 10-fold cross- validation.

**Figure 6:**
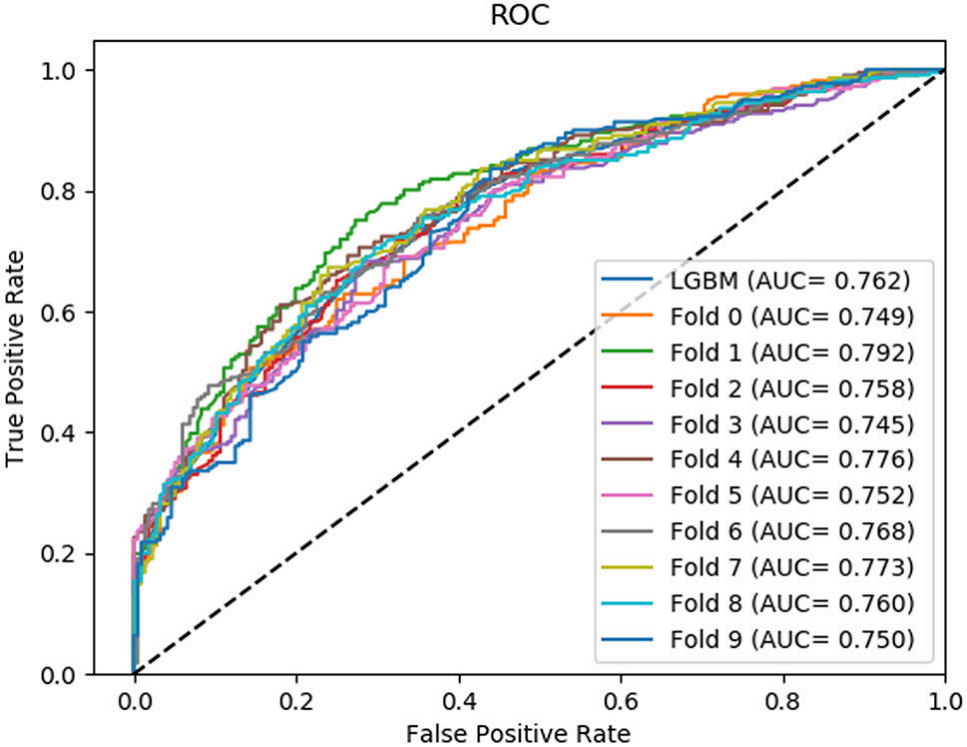
The ROC curve of dataset RPI2241 on 10-fold cross- validation.

In this section, we compared the prediction performance combined with 10-fold cross-validation of LGBM model at datasets RPI488, RPI1807, and RPI2241 with RPI-Pred, RPISeq-RF, and Inc-Pro. RPI-Pred is a SVM-based ncRNA-protein interactions prediction model proposed by Suresh et al. which based on sequence and structure information (Suresh et al., 2015). The accuracy of the RPI-Pred model on dataset RPI1807 is 93.00%. RPISeq-RF is a random forest classifier-based model proposed by Usha K Muppirala et al. which extracts feature vectors from ncRNA and protein sequence information only (Muppirala et al., 2011). And the accuracy of the RPISeq-RF model on datasets RPI488, RPI1807, and RPI2241 are 88.00, 97.30, and 63.96%, respectively. IncPro is a model proposed by Lu et al. which encodes lncRNA and protein sequences as digital vectors and scores each lncRNA-protein pair using matrix multiplication (Lu et al., 2013). Based on this IncPro model, the accuracy of datasets RPI488, RPI1807, and RPI2241 achieves 87.00, 96.90, and 65.40%, respectively. The summary comparative results of the experiments are shown in Table 5. And the 10-fold cross-validation ROC curve for our model LGBM at RPI488, RPI1807, and RPI2241 are shown in Figures 4–6.

**Table 5:** Comparison between LGBM and other methods in RPI488, RPI1807, and RPI2241.

**Dataset**	**Method**	**Accuracy (%)**	**Precision (%)**	**Sensitivity (%)**	**Specificity (%)**	**MCC (%)**
RPI488	LGBM	**89.52**	**93.28**	**94.30**	**84.17**	**79.02**
	RPISeq-RF	88.00	93.20	92.60	82.20	76.20
	IncPro	87.00	91.00	90.00	82.70	74.00
RPI1807	LGBM	96.42	**96.21**	95.20	97.40	92.76
	RPI-Pred	93.00	94.00	95.00	N/A	N/A
	IncPro	**96.90**	95.50	**96.50**	**98.10**	**93.80**
RPI2241	LGBM	**68.86**	**72.76**	**76.38**	61.50	**38.33**
	RPISeq-RF	63.96	65.37	64.83	62.59	27.98
	IncPro	65.40	66.90	65.90	**64.00**	31.00

*The bold value indicates this measure performance is the best among the compared methods*.

As shown in Table 5, our machine learning model LGBM achieved an experimental prediction accuracy of 89.52 %, higher than 88.00% of RPISeq-RF and 87.00% of IncPro on dataset RPI488. At the meantime, LGBM also had a better performance in other evaluation criterions including precision, sensitivity, specificity and MCC of 93.28, 94.30, 84.17, and 79.02%, respectively. While the performance of RPISeq-RF were 88.00, 93.20, 92.60, 82.20, 76.20% and IncPro were 87.00, 91.00, 90.00, 82.70, and 74.00%. On dataset RPI2241, except specificity, our model had a better performance of 68.86, 72.76, 76.38% on accuracy, precision and sensitivity. While on dataset RPI1807, although our experimental prediction performance was not as good as IncPro, it was still as high as 96.42, 96.21, 95.20, 97.40, and 97.26%, which is not much lower than 96.90, 95.50, 96.50, 98.10, and 93.80% of IncPro on accuracy, precision, sensitivity, specificity and MCC respectively. PRI-Pred performed slightly worse which was 93.00, 94.00, and 95.00% on accuracy, precision and sensitivity.

By comparing the prediction results, we are able to see that our prediction model LGBM has a better performance on datasets RPI488 and RPI2241, however, on dataset RPI1807, the prediction accuracy is worse than IncPro, while the accuracy is still more than 96%. In general, our model LGBM is effective and robust in predicting interactions between ncRNA and protein.

## Conclusion

In this study, we proposed an efficient prediction model LGBM using sequence and evolutionary information to predict interactions between ncRNA and protein. In order to obtain evolutionary information from protein sequences, the Zernike Moment algorithm is used to extract feature vectors of proteins from PSSM. Meanwhile, the SVD was used to extract features from k-mers sparse matrix of ncRNA, in which both the location and frequency information is preserved. On this basis, we fed the high-level feature vectors into the LightGBM classifier to predict the interaction between ncRNA and protein. To verify the accuracy and robustness of our model, 10-fold cross validation was used. Experimental results on datasets RPI369, RPI488, RPI1807 and RPI2241 demonstrated the robustness and effectiveness of our model. Therefore, the proposed LGBM model is feasible, reliable and full of generalization ability to predict ncRNA-protein interaction. Our research can be a useful tool to further biological research.

## Author contributions

Z-HZ, Z-HY, and YZ conceived the algorithm, carried out analyses, prepared the data sets, carried out experiments, and wrote the manuscript. L-PL and H-CY designed, performed and analyzed experiments and wrote the manuscript. All authors read and approved the final manuscript.

### Conflict of interest statement

The authors declare that the research was conducted in the absence of any commercial or financial relationships that could be construed as a potential conflict of interest.

## Footnotes

^1^*K-Fold Cross Validation*. Classification.
